# NKX2.5 is expressed in papillary thyroid carcinomas and regulates differentiation in thyroid cells

**DOI:** 10.1186/s12885-018-4399-1

**Published:** 2018-05-02

**Authors:** Ricardo Cortez Cardoso Penha, Luisa Aguirre Buexm, Fabiana Resende Rodrigues, Taciana Padilha de Castro, Maria Carolina S. Santos, Rodrigo Soares Fortunato, Denise P. Carvalho, Luciene C. Cardoso-Weide, Andrea C. F. Ferreira

**Affiliations:** 1 Laboratório de Fisiologia Endócrina Doris Rosenthal, IBCCF, Universidade Federal do Rio de Janeiro, Rio de Janeiro, Brazil; 2 Programa de Carcinogênese Molecular - Centro de Pesquisas (CPQ), Instituto Nacional de Câncer José Alencar Gomes da Silva (INCA), Rio de Janeiro, Brazil; 3 Departamento de Patologia, Faculdade de Medicina, Universidade Federal Fluminense, Rio de Janeiro, Brazil; 4 Centro de Estudos da Saúde do Trabalhador e Ecologia Humana- CESTEH-ENSP/FIOCRUZ, Rio de Janeiro, RJ Brazil; 5 Laboratório de Radiobiologia Molecular, IBCCF, Universidade Federal do Rio de Janeiro, Rio de Janeiro, Brazil; 6 Campus Duque de Caxias/NUMPEX, Universidade Federal do Rio de Janeiro, Rio de Janeiro, Brazil

**Keywords:** NKX2.5, Thyroid cancer, Persistence/recurrence, Papillary thyroid carcinoma, Differentiation, Iodide uptake, H_2_O_2_

## Abstract

**Background:**

NKX2.5 is a transcription factor transiently expressed during thyroid organogenesis. Recently, several works have pointed out the oncogenic role of NKX2.5 in a variety of tumors. We therefore hypothesized that NKX2.5 could also play a role in thyroid cancer.

**Methods:**

The validation of NKX2.5 expression was assessed by immunohistochemistry analysis in a Brazilian case series of 10 papillary thyroid carcinoma (PTC) patients. Then, the long-term prognostic value of NKX2.5 and its correlation with clinicopathologic features of 51 PTC patients was evaluated in a cohort with 10-years follow-up (1990–1999). Besides, the effect of NKX2.5 overexpression on thyroid differentiation markers and function was also investigated in a non-tumor thyroid cell line (PCCL3).

**Results:**

NKX2.5 was shown to be expressed in most PTC samples (8/10, case series; 27/51, cohort). Patients who had tumors expressing NKX2.5 showed lower rates of persistence/recurrence (*p* = 0.013). Overexpression of NKX2.5 in PCCL3 cells led to: 1) downregulation of thyroid differentiation markers (thyrotropin receptor, thyroperoxidase and sodium-iodide symporter); 2) reduced iodide uptake; 3) increased extracellular H_2_O_2_ generation, dual oxidase 1 mRNA levels and activity of DuOx1 promoter.

**Conclusions:**

In summary, NKX2.5 is expressed in most PTC samples analyzed and its presence correlates to better prognosis of PTC. In vitro, NKX2.5 overexpression reduces the expression of thyroid differentiation markers and increases ROS production. Thus, our data suggests that NKX2.5 could play a role in thyroid carcinogenesis.

**Electronic supplementary material:**

The online version of this article (10.1186/s12885-018-4399-1) contains supplementary material, which is available to authorized users.

## Background

The homeodomain transcription factor NKX2.5 is critical for differentiation and proliferation of the primitive pharyngeal endodermal cells [1], being expressed up to the embryonic day 11.5 in the mouse thyroid primordium [2]. However, NKX2.5 expression is discontinued just before the appearance of proteins that are crucial for thyroid hormone biosynthesis, such as thyroglobulin (Tg), thyroperoxidase (TPO), thyrotropin receptor (TSHR), sodium-iodide symporter (NIS) and dual oxidases (DuOx1 and 2) [2–4].

Even though NKX2.5 expression seems to be restricted to heart in adults [5], NKX2.5 has been described to be expressed in several types of tumors, including pediatric acute lymphoblastic leukemia [6], skin squamous cell carcinoma [7] and ovarian yolk sac tumor [8]. However, the possible role of NKX2.5 in thyroid cancer remains elusive.

Thus, in the present work, we aimed at evaluating NKX2.5 expression by immunohistochemistry in 10 PTC samples. To assess NKX2.5 association with clinical prognostic, we have also included 51 patients from a well-characterized 10 years Brazilian cohort of PTC patients [9]. We have also investigated the functional role of this transcription factor on the expression of thyroid differentiation markers and reactive oxygen species (ROS) production in normal thyroid cells (PCCL3). To our knowledge, this is the first study showing NKX2.5 expression in PTC samples.

## Methods

### Patients

To validate the expression of NKX2.5, we have included 10 patients admitted to the hospital in 2012, with the diagnosis of PTC, according to the International Classification of Diseases for Oncology (ICD-O) 80,503 (papillary thyroid carcinoma) in the register of the Cancer Hospital from the centre’s database. The eligible patients were submitted to thyroid surgery to treat PTC and were aged 18 years or more at the time of surgery.

To assess NKX2.5 association with clinical prognostic, we have also included 51 patients [9] admitted to the hospital between January 1st, 1990, and December 31st, 1999, with a diagnosis of PTC as per the International Classification of Diseases for Oncology (ICD-O) codes 83,403, 82,603 or 80,503 (follicular variant of PTC, thyroid papillary adenocarcinoma (SOE), papillary thyroid carcinoma, respectively) in the register of the Cancer Hospital from the centre’s database, which was available in 2010. The eligible patients had undergone thyroidectomy as a type of thyroid surgical approach to treat PTC in the study period and were aged 18 years or more at time of initial surgery.

The three main outcome variables of interest were persistence, recurrence and PTC-free status. For this classification, we have only considered the first event experienced by the patient. Another outcome studied was death as a result of PTC and/or from any other cause. PTC persistence was defined as evident residual disease (active disease) until 12 months after initial surgical treatment. Furthermore, PTC recurrence was defined as having the first event of active disease occurring between 1 and 10 years of follow-up. Patients were considered PTC-free if they did not show active disease after the initial surgery, in the period between the initial surgery and ten years of follow-up. Active PTC disease was defined when one or more of the following was observed: (a) structural disease evidenced by positive imaging findings or after radioactive iodine (^131^I, RAI) therapy; and (b) biochemical evidence of disease, with significant increase in serum thyroglobulin (Tg) levels during thyroid hormone treatment (levothyroxine, LT4) compared with previously stable levels and/or an increase in serum Tg levels after LT4 withdrawal (stimulated Tg). The clinicopathologic information in each case, including age, gender, treatment, pathologic stage and presence or absence of tumor recurrence or persistence, was obtained from medical records and tumor registries. The characteristics of the 51 patients selected for this study are summarized in Table 1.

**Table 1:** Clinicopathologic characteristics of PTC patients (*n* = 51)

Variable	Category	No. of cases	% of cases
*Gender*	Male	10	19.6
	Female	41	80.4
*Age*	≤40 years	23	45.1
	41–60 years	11	21.6
	> 60 years	17	33.3
*Type of surgery*	Total or near total thyroidectomy	42	82.4
	Partial thyroidectomy	7	13.7
	Others	2	3.9
*Neck dissection*	Yes	27	52.9
	No	24	47.1
*Tumour size*	≤4 cm	43	84.3
	> 4 cm	8	15.7
*Pathological stage of primary tumor size (pT)*	pT1 and pT2	22	43.1
	pT3 and pT4	28	54.9
	pTx	1	2.0
*Pathological stage of regional lymph node (pN)*	pN1	30	58.8
	pN2	3	5.9
	pNx	18	35.3
*Pathological stage of distant metastasis (pM)*	pM0	42	82.4
	pM1	9	17.6
*Pathological stage (pTNM)*	pTNM I	22	43.1
	pTNM II	3	5.9
	pTNM III	4	7.8
	pTNM IV	15	29.4
	Unknown	7	13.8
*Histopathological classification*	Classic papillary carcinoma	40	78.2
	Follicular variant	5	9.8
	Tall cell variant	2	4.0
	Clear cell variant	1	2.0
	Solid variant	1	2.0
	Oncocytic variant	1	2.0
	Others	1	2.0
*Extrathyroid extravasation*	Yes	30	58.8
	No	18	35.3
	Unknown	3	5.9
*Vascular or angiolymphatic invasion*	Yes	23	45.1
	No	22	43.1
	Unknown	6	11.8
*Morbidity outcome*	Without recurrence or persistence	21	41.2
	Recurrence	9	17.6
	Persistence	21	41.2
*Place of outcome*	Without recurrence or persistence	21	41.2
	Local and/or regional lesion only	11	21.6
	Distant metastasis only	8	15.7
	Both (Local and regional lesion and distant metastasis)	10	19.6
	Unknown	1	1.9
*Lethality within 10 years follow-up*	Yes	8	15.7
	No	43	84.3
*Cause of death*	PTC	6	75
	Others	2	25

### Immunohistochemistry analysis

Immunohistochemistry (IHC) analysis was performed on paraffin sections of the papillary thyroid carcinomas mounted on glass slides. Tissue sections of heart necropsy, obtained from the “Departamento de Patologia da Universidade Federal Fluminense”, served as NKX2.5 positive control. For antigen retrieval, the slides were incubated in a pH 6.0 solution (target antigen retrieval solution) for 45 min in a water bath, at 96 °C, followed by a washing step with phosphate-buffered saline (PBS). Incubations with the primary antibody against NKX2.5 (polyclonal anti-NKX2.5, SAB2101601, Sigma-Aldrich; diluted 1:800) were performed overnight at 4 °C. Samples were then incubated with biotinylated secondary antibodies using the streptavidin-biotin-peroxidase kit (Strep ABC complex/HRP Duet kit, DAKO Cytomation). The reactions were developed with a solution containing diaminobenzidine tetrahydrochloride chromogen, and the sections were counterstained with Harris’s hematoxylin. Negative and positive controls were included in all reactions.

All the sections were assessed independently by two pathologists, who met to resolve discordant interpretations and establish a consensus categorization. A binary classification (positive vs. negative) was used to score the IHC. The positive slides were evaluated semi-quantitatively by the distribution of the immunohistochemical positivity (1–49% and 50–100%) of neoplastic cells. Whenever the distribution was < 50%, the cases were classified as low expression, and cases with ≥50% of cells stained were classified as high expression. Positive slides were also classified according to the subcellular distribution of NKX2.5 in cytoplasmic and nuclear.

### Cell culture

The non-tumoral rat thyroid cell line (PCCL3) was kindly donated by dr. Fusco, from the Department of Molecular Medicine and Medical Biotechnology, Naples University, who developed this cell line. PCCL3 was maintained in Coon’s modified Ham’s F-12 medium (HiMedia Laboratories, Mumbai, India), supplemented with 5% FBS and a six-hormone mixture (1 mU/ml TSH, 10 μg/ml insulin, 5 μg/ml transferrin, 10 nM hydrocortisone, 10 ng/ml somatostatin, and 10 ng/ml glycyl-L-histidyl-L-lysine acetate) and maintained in a humidified 5% CO_2_ incubator at 37 °C, as previously described [10]. The highly transfectable cell line derivate from human embryonic kidney 293 cells (HEK293T) were grown in Dulbecco’s modified Eagle’s medium (DMEM) (HiMedia Laboratories, Mumbai, India), supplemented with 10% FBS.

### Transient transfection assays

#### NKX2.5 overexpression

The *NKX2.5* plasmid (7036 bp) (p*NKX2.5*) contains the full length of *NKX2.5* coding region (RefSeq NM_008700), cloned into pcDNA3.1 expression vector (Invitrogen, Carlsbad, California) [11]. PCCL3 cells (1.2 × 10^5^) were seeded in 24-well plate and 1 μg of p*NKX2.5* or pcDNA3.1 (empty vector, control) were transfected, using Lipofectamine LTX combined with PLUS reagent (Invitrogen, Carlsbad, California) diluted in Ham’s F-12 complete medium. All procedures were performed following the manufacturer’s recommendations.

The efficiency of the transient transfection was evaluated 24 h later by real time PCR with specific oligonucleotides for *NKX2.5* gene and immunoblotting analysis using NKX2.5 primary antibody (SAB2101601-Sigma-Aldrich, St. Louis, MO, USA). Concomitantly, PCCL3 cells were transfected with the vector encoding the green fluorescent protein, in order to confirm the transfection efficiency, using the same procedure described above.

#### Real time PCR

Total RNA from cell line was extracted using the RNeasy® Plus Mini Kit (Qiagen, Valencia, California), following the manufacturer’s instructions and subsequently quantified by NanoVue™ Plus spectrophotometer (GE Healthcare, Sweden). Total RNA (0.5–1 μg) was reversely transcribed using MultiScribe™ Reverse Transcriptase (Applied Biosystems, Foster City, CA), in accordance to the manufacturer’s instructions. Reactions for the quantification of mRNA by real-time PCR were performed in an ABI Prism 7500 Sequence Detection System from Applied Biosystems, using 6 μl Maxima SYBR Green qPCR Master Mix (Thermo Scientific, Rockford, IL, USA), 0.5 μl specific oligonucleotides (150 nM), 2.5 μl DEPC water and 3 μl diluted cDNA. The oligonucleotides for real-time PCR were purchased from Applied Biosystems, designed with PrimerQuest software (Integrated DNA Technologies, San Diego, CA, USA) and are listed in Additional file 1. *RPL4* was used as internal control. Relative gene expression was determined by subtracting cycle threshold (CT) for the gene of interest from CT for the reference gene, calculated using the 2^-ΔΔCT^ method, as previously described [12] and expressed as relative to control.

#### Western blot

Cells were homogenized in lysis buffer containing 135 mM NaCl, 1 mM MgCl_2_, 2.7 mM KCl, 20 mM Tris, pH 8.0, 1% Triton, 10% glycerol and protease and phosphatase inhibitors (0.5 mM Na_3_VO_4_, 10 mM NaF, 1 mM leupeptin, 1 mM pepstatin, 1 mM okadaic acid, and 0.2 mM phenylmethylsulfonyl fluoride), and then syringed five times. An aliquot was used to determine the concentration of protein by BCA protein assay kit (Pierce, Rockford, IL, USA), as recommended by the manufacturer. Samples were then subjected to SDS/PAGE electrophoresis, transferred to PVDF membranes, and probed with the following antibodies: 1:2000 polyclonal anti-NKX2.5, Sigma-Aldrich; 1:4000 monoclonal anti-GAPDH, Millipore; 1:2000 anti-rabbit IgG HRP-linked antibody and 1:4000 anti-mouse IgG HRP-linked antibodies from Cell Signaling. Detection of the proteins was performed using ECL (Thermo Scientific, Rockford, IL, USA).

#### Cell viability assay

As an index of cell viability, we used the commercially available MTT assay (Sigma-Aldrich, St. Louis, MO, USA), according to the recommendations of the manufacturer. The assay is based on the cellular conversion of the tetrazolium salt into formazan that is soluble in culture medium and is directly measured at 490 nm, in a 96-well plate, using a spectrophotometer. Absorbance is directly proportional to the number of living cells in culture. PCCL3 cells were transfected with pcDNA3.1 (control) or p*NKX2.5* and 0, 24, 48 and 72 h later MTT assay was performed. Cells were incubated with MTT (0.5 mg/ml) for 3 h at 37 °C in a humidified 5% CO_2_ atmosphere. Then, cells were lysed with DMSO (PA). All determinations were made in triplicates and the results were expressed as relative to pcDNA3.1 in initial time (0 h).

#### Iodide uptake assay

Iodide uptake assay was performed as described by Souza et al. [10]. Briefly, PCCL3 cells (1 × 10^5^) were grown in 24-well plates, transfected with pcDNA3 empty vector or p*NKX2.5*, as described above, and 24 h later they were incubated for 45 min at 37 °C in 1 ml Hank’s balanced salt solution (HBSS) containing 0.1 μCi carrier-free Na^125^I and 100 μM NaI. For each experimental condition, a well also received 10 μM KClO_4_, a competitive inhibitor of *NIS*, in order to determine the nonspecific radioiodide uptake. After incubation, cells were washed once with ice-cold HBSS and lysed with 0.1 M NaOH, and the radioactivity was determined in a gamma counter (Compu-Gama, 1214, LKB Wallac). An aliquot of each sample was used to determine the protein concentration, using BCA protein assay kit (Pierce, Rockford, IL, USA), as recommended by the manufacturer. Specific iodide uptake value was obtained by subtracting iodide uptake in the absence and in the presence of KClO_4_ and related to protein concentration. Results were expressed as specific units of iodide accumulation relative to control.

#### Extracellular H_2_O_2_ production

Extracellular H_2_O_2_ generation was quantified by the Amplex red/horseradish peroxidase assay, which detects the accumulation of a fluorescent oxidized product, as previously described [13]. 24 h after transfection with pcDNA3.1 or p*NKX2.5*, PCCL3 cells (1 × 10^5^) were incubated in Dulbecco’s PBS (D-PBS) containing CaCl_2_, MgCl_2_, D-glucose (1 mg/ml), ionomycin (1 μM), superoxide dismutase (100 U/ml), horseradish peroxidase (0.5 U/ml), and Amplex red (50 μM), and the fluorescence was immediately measured in a microplate reader (VictorX4) for 30 min (excitation wavelength = 530 nm and emission wavelength = 595 nm). Hydrogen peroxide concentration was determined using standard calibration curves and the result was expressed as nmol H_2_O_2_ per hour per 10^5^ cells. The measurements were made in the presence and in the absence of ionomycin, a calcium ionophore, since dual oxidases are calcium-dependent enzymes, and H_2_O_2_ generation obtained in the presence of ionomycin were subtracted from that obtained in the absence of ionomycin.

#### DuOx1 promoter activity

For gene reporter assay, we used the *DuOx1* promoter (pDuOx1) plasmid (1 μg), containing the proximal 5′-flanking region of *DuOx1* gene and the luciferase reporter gene cloned in the PgL3 vector [14]. It was transiently transfected in combination with wild type or mutated *NKX2.5* (Ile^183^➔Pro) expression vectors [11] or the corresponding empty vector, pcDNA3.1 (500 ng) in HEK293T cells (2 × 10^5^ cells/well), using Lipofectamine LTX combined with PLUS reagent (Invitrogen, Carlsbad, California), as described above. pRL-CMV, which contains renilla cDNA, was used to correct for transfection efficiency (Promega, Madison, WI, USA). After 24 h, cells were harvested and collected for luciferase and renilla activity by the Dual-Luciferase reporter assay system (Promega, Madison, WI, USA). Luminescence was measured in a Victor X4 Multilabel Plate Reader (PerkinElmer, Norwalk, CT, USA). Results were expressed as relative activity, compared to the control (pcDNA3.1) in each experiment.

#### Statistical analysis

For the statistical analysis, we used the R program (Free Software Foundation, USA) and commercially available software SPSS 20.0 (SPSS Inc., Chicago, IL). Descriptive statistics were used in a preliminary analysis of the relation between baseline variables and outcome events. The PTC-free group was considered as the reference group. The association between the clinic pathological variables and NKX2.5 immunoexpression were analyzed by the Fisher’s exact tests. In addition, the Kaplan-Mayer method was used to evaluate whether the presence of *NKX2.5* impacts on morbidity outcomes (persistence/recurrence) and lethality. Tests were considered statistically significant when the *p*-value was < 0.05.

All in vitro results were expressed as mean ± SEM and were analyzed by the non-parametric Mann Whitney’s test (when comparing two groups) or by the non-parametric Kruskal-Wallis test followed by Dunn’s multiple comparison test (when comparing three or more groups). Statistical analyses were performed using the software Graphpad Prism (Version 5, Graphpad Software Inc., San Diego, USA) and the difference was considered significant when *p* < 0.05.

## Results

### NKX2.5 is expressed in PTC samples

**Fig. 1:**
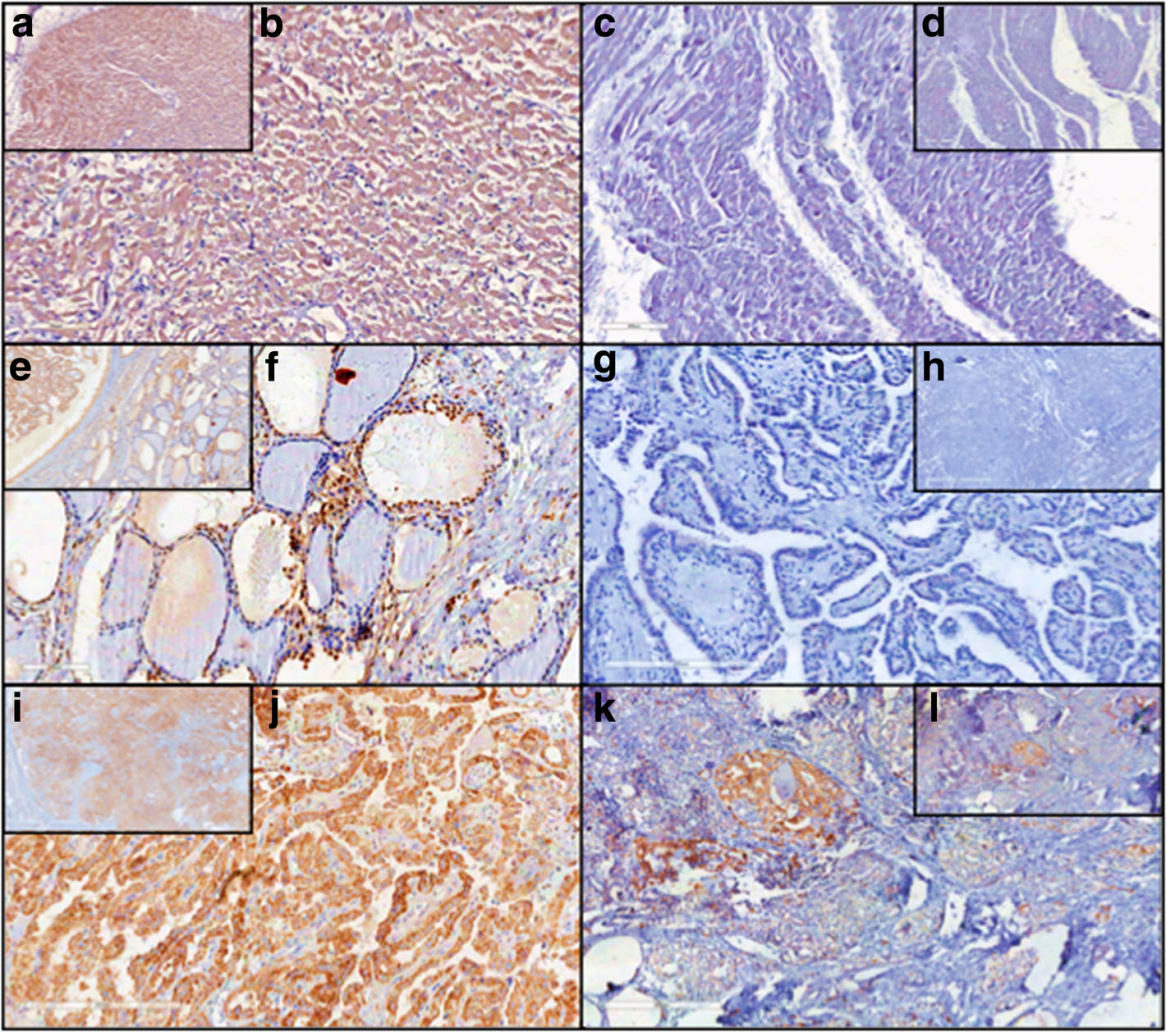
Panel with immunohistochemical expression of NKX2.5: (**a**), (**b**) Human heart tissue - positive control with nuclear and cytoplasmic immunostaining (*10X, 20X*); (**c**), (**d**) Human heart tissue - negative control without immunostaining (*5X, 10X*); (**e**), (**f**) Adjacent normal thyroid tissue - nuclear immunostaining (*10X, 20X*); (**g**), (**h**) Papillary thyroid carcinoma - absence of immunostaining (*2X, 20X*); (**i**), (**j**) Papillary thyroid carcinoma - high cytoplasmic immunostaining (*2X, 20X*); and (**k**), (**l**) Papillary thyroid carcinoma - low cytoplasmic immunostaining (*5X, 10X*)

As expected, NKX2.5 was shown to be expressed in human heart, both in cytoplasm and nucleus (Fig. 1a and b), but not in negative control heart (without primary antibody, Fig. 1c and d), what is in accordance to literature data [5]. On the other hand, even though NKX2.5 was described to be transiently expressed only during thyroid organogenesis, until embryonic day 11.5 in mouse ^2^, normal human thyroid tissue, adjacent to papillary thyroid carcinoma, showed a marked nuclear staining for this transcription factor (Fig. 1e and f), suggesting that *NKX2.5* might be re-expressed later in life or that the proximity to cancer environment had an impact on the pattern of expression of neighbor cells.

In papillary thyroid carcinoma, we found variable degrees of NKX2.5 expression, with some patients showing no expression (Fig. 1g and h), while others showed high (Fig. 1i and j) or low (Fig. 1k and l) cytoplasmic expression. Differently from paranodular tissue, NKX2.5 staining in thyroid papillary carcinoma, when present, was predominantly found in cytoplasm. Data from all 10 patients are summarized in Additional file 2. Most cases of thyroid papillary carcinoma (8/10) were positive for NKX2.5.

### Overexpression of NKX2.5 promotes a dedifferentiation phenotype in thyroid cells

**Fig. 2:**
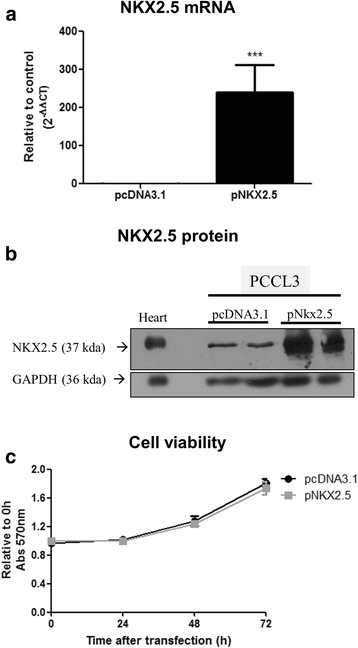
NKX2.5-overexpressing PCCL3 cell viability**.** The cells were transfected with empty vector (pcDNA3.1) or with the plasmid encoding for *NKX2.5* (p*NKX2.5*) and overexpression was confirmed by (**a**) mRNA (n = 7 per condition; *** p < 0.001 vs. pcDNA3.1) and (**b**) protein levels (*n* = 2). (**c**) Cell viability, evaluated by MTT assay, performed 0, 24, 48 and 72 h after transfection (*n* = 8 per condition). GAPDH was used as loading control for Western blot analysis and *RPL4* for RT-PCR

In order to investigate the role of NKX2.5 on thyroid function, we overexpressed it in a non-tumoral rat thyroid cell line (PCCL3), which led to an increment in both *NKX2.5* mRNA and protein levels (Fig. 2a and b), but did not affect cell viability (Fig. 2c). Even though PCCL3 cells are of rat origin, they were chosen to evaluate the effect of NKX2.5 overexpression because PCCL3 cells keep in vitro important markers of thyroid differentiation (such as ability to trap iodide, synthesize thyroglobulin and TSH-dependency for growth) and they have the same chromosome number (42) of normal rat tissues [15].

**Fig. 3:**
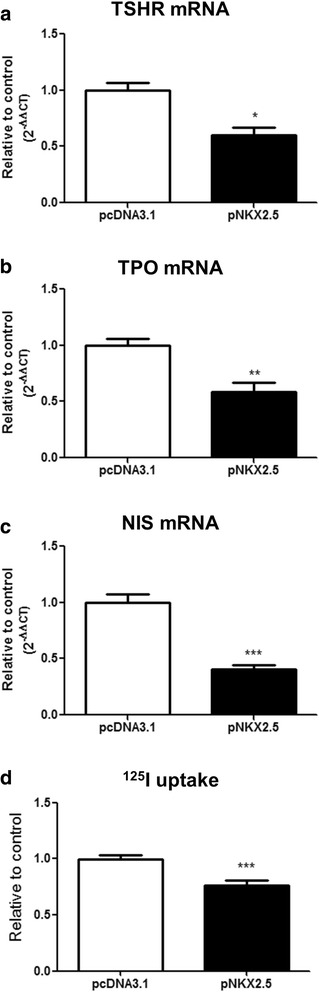
Overexpression of NKX2.5 reduces thyroid differentiation markers and iodide uptake in PCCL3 cells: PCCL3 cells were transfected with empty vector (pcDNA3.1) or with the plasmid encoding for *NKX2.5* (pNKX2.5) and, 24 h later, we have evaluated: (**a**) *TSH receptor* (*TSHR*), (**b**) *Thyroperoxidase* (*TPO*) and (**c**) *Sodium-iodide symporter* (*NIS)* mRNA levels, besides (**d**) iodide uptake (for RT-PCR, *n* = 7 per condition and for iodide uptake assay, *n* = 9 per condition; * p < 0.05 vs. pcDNA3.1, ** p < 0.01 vs. pcDNA3.1, ***p < 0.001 vs. pcDNA3.1). Data are expressed as relative to control (empty vector - pcDNA3.1). TSHR – thyrotropin receptor; TPO – thyroperoxidase; NIS – sodium-iodide symporter

Transfection of PCCL3 cells with wild-type *NKX2.5* (pNKX2.5) induced a significant reduction of TSH receptor (*TSHR*) (Fig. 3a) and thyroperoxidase (*TPO*) (Fig. 3b) mRNA levels, which are important thyroid differentiation markers [16]. Additionally, overexpression of NKX2.5 decreased *NIS* mRNA levels (Fig. 3c), leading to a reduced iodide uptake in PCCL3 cells (Fig. 3d). Therefore, NKX2.5 seems to have a negative impact in the expression of proteins that are important for thyroid hormone synthesis. These results are in agreement with the disappearance of NKX2.5 just before the beginning of thyroid hormonogenesis [2].

### Differential regulation of dual oxidases by NKX2.5 in PCCL3 cells

**Fig. 4:**
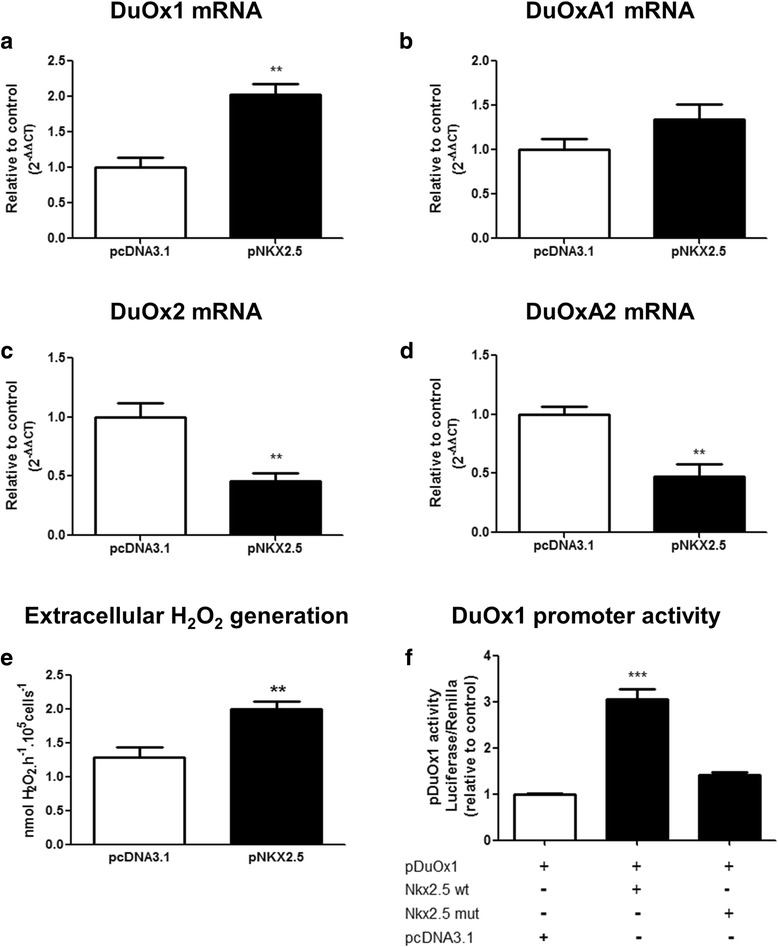
Effect of NKX2.5 on the expression and activity of DuOx: PCCL3 cells were transfected with empty vector (pcDNA3.1) or with the plasmid encoding for *NKX2.5* (pNKX2.5) and we have evaluated mRNA levels of the following genes: (**a**) *DuOx1*, (**b**) *DuOxA1*, (**c**) *DuOx2* and (**d**) *DuOxA2*, as well as (**e**) extracellular H_2_O_2_ generation (for RT-PCR, *n* = 7 per condition and for DuOx activity, *n* = 6 per condition; ** *p* < 0.01 vs. pcDNA3.1); (**f**) Wild type *NKX2.5* (Nkx2.5 wt) and mutant *NKX2.5* (Ile^183^ to Pro mutation - *Nkx2.5 mut*) were transfected in HEK293T cells and the activity of pDuOx1 (*DuOx1* promoter) was evaluated. Empty vector pcDNA3.1 was transfected as control. Renilla was co-transfected in HEK293T cells to normalize luciferase activity. All transfections were done in triplicate at least twice (*n* = 6 per condition). *** *p* < 0.001 vs. pcDNA3.1. Data are expressed as relative to control (empty vector - pcDNA3.1). DuOx1 – dual oxidase 1; DuOx2 – dual oxidase 2; DuOxA1 - dual oxidase maturation factor 1; DuOxA2 - dual oxidase maturation factor 2

Besides iodide uptake and TPO activity, hydrogen peroxide generation is also important for thyroid hormone biosynthesis [4]. Dual oxidases (DuOx1 and DuOx2) are responsible for thyroid H_2_O_2_ generation and the complete glycosylation and activity of these enzymes require the expression of their maturation factors DuOxA1 and DuOxA2 [17]. Thus, we have next decided to study the effect of NKX2.5 overexpression on DuOx expression and activity in PCCL3 cells. In fact, NKX2.5 overexpression in PCCL3 resulted in increased *DuOx1* mRNA levels (Fig. 4a), while the maturation factor *DuOxA1* mRNA remained unchanged (Fig. 4b). On the other hand, NKX2.5 overexpression down regulated both *DuOx2* (Fig. 4c) and *DuOxA2* mRNA levels (Fig. 4d). In addition, extracellular H_2_O_2_ generation was stimulated by NKX2.5 (Fig. 4e), in agreement with increased *DuOx1* mRNA levels, which is known to be the main DuOx isoform in PCCL3 cells [18]. It is noteworthy that *NOX4* mRNA was not detected in PCCL3 cells in our experimental conditions (data not shown).

Since *NKX2.5* transfection increased *DuOx1* expression and activity in PCCL3 cells, we decided to evaluate whether this effect could occur at the transcriptional level. Thus, we studied the effect of NKX2.5 (wild type or mutant) overexpression on *DuOx1* promoter. As shown in fig. 4f, NKX2.5 stimulated *DuOx1* promoter (pDuOx1) when compared to basal promoter activity. Moreover, transfection of Ile^183^➔Pro NKX2.5 mutant (*Nkx2.5* mut), which keeps homodimerization function but does not bind DNA [11], did not have the same effect (Fig. 4f), suggesting that interaction with DNA is essential for *DuOx1* promoter regulation by NKX2.5. Thus, the effect of NKX2.5 regulating DuOx1 seems to occur at the transcriptional level.

### The expression of *NKX2.5* predicts better prognosis in PTC samples

We then wondered whether the expression of NKX2.5 could impact on the outcome of the patients with PTC. The study population was predominantly composed of females (89.4%), with a mean age of 48 years (ranging from 18 to 81 years). Most patients underwent surgical treatment of total or near total thyroidectomy (82.4%). Tumors smaller than 4 cm (84.3%) and in pathological stage I (43.1%) were predominant. Twenty-one (41.2%) cases had persistent disease and nine cases (17.6%) with recurrence were identified. According to histopathological classification, classic papillary carcinomas (78.7%) were predominant. Eight out of 51 cases (15.7%) died as a result of PTC and/or from any other cause, according to the cancer death registry of the hospital (Table 1). The expression of NKX2.5 was observed in 53% of the cases, being 31.4% with low score in the tumor (Table 2). The presence of NKX2.5 in the primary thyroid tumor was not associated to lethality or morbidity in 10 years. According to the outcome, negative score of NKX2.5 was related to persistence (*p* = 0.013) and pathological stage of primary tumor sizes pT1 and pT2 (*p* = 0.029). All the details about the association between NKX2.5 expression and patients’ data are provided in Table 2. Thus, our data suggest that the expression of NKX2.5 in PTC samples is associated to a better prognosis for the patients.

**Table 2:** Clinicopathologic characteristics and immunohistochemical expression of NKX2*.5* of the PTC tumor (*N* = 51). Data were analyzed by Fisher’s exact test

Variable	Category	Score of NKX2.5			
		Negative No. (%)	Low No. (%)	High No. (%)	*p* Value
*NKX2.5 expression*	Negative	24 (47.1%)	0 (0.0%)	0 (0.0%)	
	Positive	0 (0.0%)	16 (31.4%)	11 (21.6%)	
*Outcome*	Without recurrence or persistence	7 (13.7%)	6 (11.8%)	8 (15.7%)	*0.013*
	Recurrence	3 (5.9%)	6 (11.8%)	0 (0.0%)	
	Persistence	14 (27.5%)	4 (7.8%)	3 (5.9%)	
*Pathological stage of primary tumor size (pT)*	pT1 and pT2	14 (27.5%)	3 (5.9%)	6 (11.8%)	*0.029*
	pT3 and pT4	10 (19.6%)	13 (25.5%)	4 (7.8%)	
	pTx	0 (0.0%)	0 (0.0%)	1 (2.0%)	

Negative score of NKX2.5 was related to persistence (*p* = 0.013) and pathological stage of primary tumor sizes pT1 and pT2 (*p* = 0.029)

## Discussion

The regulation of cell proliferation and differentiation is crucial for both normal development and carcinogenesis, therefore it is expected that genes critical for thyroid organogenesis could also play a role in thyroid cancer [19]. In this study, we reported that NKX2.5 is highly expressed in PTC samples. The expression of NKX2.5 has been described in a variety of tumors, being correlated to a malignant transformation of the neoplastic cells [6–8]. In agreement with that, we have observed a nuclear immunostaining of NKX2.5 in normal adjacent thyroid tissue, which suggest that the expression of this transcription factor could be an early event of thyroid carcinogenesis. Accordingly, our data revealed that NKX2.5 overexpression decreased mRNA levels of the thyroid differentiation markers (*TPO, NIS* and *TSH receptor*) in normal thyroid cells, which reinforces our hypothesis that NKX2.5 promotes a dedifferentiation phenotype in thyroid cells. Above all, we have found a reduction in iodide uptake, which could be due to oxidative damage of NIS, since NKX2.5 overexpression induced an increment in hydrogen peroxide generation, and/or to a direct effect of NKX2.5 down regulating NIS. It is important to underline that NIS regulation is of great relevance not only for thyroid physiology but also for the management of thyroid diseases, since radioiodine therapy is used to treat thyroid cancer and the loss of NIS is associated with poor prognosis of thyroid cancer patients [20, 21].

Elevated amounts of ROS have been related to genomic instability and tumorigenesis in thyroid cells [22] and H_2_O_2_-generating activity of DuOx1 seems to contribute to these events [23]. Overexpression of NKX2.5 in PCCL3 cells resulted in enhanced H_2_O_2_ generation, which seems to be due to increased DuOx1 activity, since mRNA levels of *DuOx1* were up regulated by NKX2.5 and *NOX4* mRNA was not detected in PCCL3 in our assay conditions. Even though *DuOx2* and *DuOxA2* mRNA levels were reduced in cells transfected with *NKX2.5*, it is well known that *DuOx1* is the main source of H_2_O_2_ in PCCL3 cells [18]. Interestingly, our results suggest that NKX2.5 upregulates *DuOx1* at the transcriptional level, leading to increased ROS production and thus reinforcing the idea that NKX2.5 could predispose thyroid cells to carcinogenesis.

Herein, we have found a predominantly cytoplasmic expression of NKX2.5 in PTC samples. NKX2.5 activation and subcellular localization have been shown to be regulated by factors such as matrix stiffness and sumoylation [24, 25]. Since sumoylation machinery, which is known to activate NKX2.5 [25], is reduced in PTC [26], we hypothesize that the cytoplasmic localization of NKX2.5 in PTC could be due to a reduction in NKX2.5 sumoylation.

Changes in DNA methylation have been shown to induce tumor initiation and progression [27], and PTC exhibits global hypomethylation when compared to normal thyroid [28]. Interestingly, the expression of NKX2.5 is regulated by methylation of the promoter region [29] and hence, an epigenetic dysregulation of *NKX2.5* promoter region might be a plausible mechanism underlying NKX2.5 overexpression in PTC samples.

Although the vast majority of PTC patients has good prognosis, 1/3 of all cases persist or relapse [30]. In our study, the absence of NKX2.5 was associated to the persistence of PTC, suggesting that NKX2.5 expression does not seem to contribute to clinic aggressiveness of the disease. Furthermore, treated patients who express NKX2.5 have lower rate of persistence/recurrence, indicating that its expression might be associated to a less aggressive tumor behavior. So, NKX2.5 could be useful as a molecular target to help predicting the outcome of PTC patients, clinically relevant information to decide the best therapeutic approach. Moreover, in PTC patients, the absence of NKX2.5 was associated to the pathological stage of primary tumor size pT1 and pT2, despite the fact that PCCL3 cell viability was not affected by *NKX2.5* transfection. Thus, in tumor cells, NKX2.5 might impact cell proliferation and/or survival. In fact, NKX2.5 has been suggested to have a role enhancing survival of leukemic T-cells [31].

Our data suggest that NKX2.5 has a dual role in thyroid. During thyroid organogenesis NKX2.5 seems to be important during the beginning of the organogenesis, but latter, NKX2.5 have to disappear before thyroid differentiation markers can be expressed [2]. In a similar way, during thyroid carcinogenesis, NKX2.5 might play a role during tumour initiation, inducing dedifferentiation, as shown in PCCL3 overexpressing NKX2.5. However, thyroid cancer progression might require the disappearance of NKX2.5, thus explaining the correlation between the levels of NKX2.5 expression and the better prognosis. The mechanisms underlying this shift in NKX2.5 expression during thyroid cancer progression could involve many factors, such as changes in NKX2.5 sumoylation, which is known to regulate NKX2.5 [25], changes in the tumour microenvironment, changes in the expression of other transcription factor that could regulate NKX2.5, increased expression of miRNA targeting NKX2.5, among others. Literature data have shown low frequency of BRAF mutations in distant metastases, in comparison with the paired primary tumours [32], thus suggesting that this mutation could play a role in thyroid cancer initiation but not progression. Thus, NKX2.5 could have a similar effect, contributing for thyroid cancer initiation but not for progression.

## Conclusions

We have reported for the first time that NKX2.5 is expressed in PTC and regulates differentiation, iodide uptake and extracellular ROS generation in thyroid cells. Our findings also unravel NKX2.5 as promising molecular target that could help to predict patients’ outcomes.

## Additional files

Additional file 1: **Table S1.** Primers used for Real Time PCR assay. (DOC 32 kb)

Additional file 2: **Table S2.** Immunohistochemical analysis of NKX2.5 in papillary thyroid carcinoma samples (*n* = 10). (DOC 29 kb)

## Abbreviations

CT: Cycle threshold
DuOx: Dual oxidase
NIS: Sodium-iodide symporter
PBS: Phosphate-buffered saline
PTC: Papillary thyroid carcinoma
RAI: Radioactive iodine
ROS: Reactive oxygen species
Tg: Thyroglobulin
TPO: Thyroperoxidase
TSHR: Thyrotropin receptor
