# Supplementary material for: NKX2.5 is expressed in papillary thyroid carcinomas and regulates differentiation in thyroid cells
Source: BMC Cancer. 2018 May 2;18:498. doi: 10.1186/s12885-018-4399-1 (PMC5930850; doi:10.1186/s12885-018-4399-1)
Supplement: Supplementary file 1 — Table S1. Primers used for Real Time PCR assay. (DOC 32 kb) [file 12885_2018_4399_MOESM1_ESM.doc]

**Additional file 1**

**Table 1:** Primers used for Real Time PCR assay.

| NKX2.5 Forward  NKX2.5 Reverse | 5’-CTC CAG AGT CTG GTC CTG CCG-3’  5’-CCT GCA TGC TGG CCG CCT TC-3’ |
| --- | --- |
| DuOx1 Forward  DuOx1 Reverse | 5’-CTC ATC CTG TCC AGC AAC C-3’  5’-GTC CAG TCC ATT CTC CTT CAG-3’ |
| DuOxA1 Forward  DuOxA1 Reverse | 5’-TGA CCA GCT TAT TCA TCG GG-3’  5’-CTG TGA GGG TGA TGT TGA GTC-3’ |
| DuOx2 Forward  DuOx2 Reverse | 5’-TGC TCT CAA CCC CAA AGT G-3’  5’-TCT CAA ACC AGT AGC GAT CAC-3’ |
| DuOxA2 Forward  DuOxA2 Reverse | 5’-TGG TAT TCT TGT CCT TGG CTG-3’  5’-GGA GGT ACT GAA GGC TTT GTA G-3’ |
| NIS Forward  NIS Reverse | 5’-GCC CCA AAG GAA GAC ACT G-3’  5’-CAT CGT GCC CCA GAT ACA G-3’ |
| TPO Forward  TPO Reverse | 5’-GAA TGA GGA ACT GAC CGA GAG-3’  5’-TGA CAA GCC ACA GAA CTC TC-3’ |
| TSHR Forward  TSHR Reverse | 5’-AGG TCC CTT GGA AAA ATG AGG-3’  5’-GTC TCG ATG AGC TTC AGA GTC-3’ |
| RPL4 Forward  RPL4 Reverse | 5’-GAT GAA TTGTAC GGC ACT TGG-3’  5’-TCT TTG GAT CTC TGG GCT TTT TC-3’ |
