# Supplementary material for: NKX2.5 is expressed in papillary thyroid carcinomas and regulates differentiation in thyroid cells
Source: BMC Cancer. 2018 May 2;18:498. doi: 10.1186/s12885-018-4399-1 (PMC5930850; doi:10.1186/s12885-018-4399-1)
Supplement: Supplementary file 2 — Table S2. Immunohistochemical analysis of NKX2.5 in papillary thyroid carcinoma samples (n = 10). (DOC 29 kb) [file 12885_2018_4399_MOESM2_ESM.doc]

**Additional file 2**

**Table 2**: Immunohistochemical analysis of NKX2.5 in papillary thyroid carcinoma samples (n=10).

| **NKX2.5** |  | **No.** |
| --- | --- | --- |
| **Expression** | *Positive* | 8/10 |
|  | *Negative* | 2/10 |
| **Score of positive cases** | *High* | 6/8 |
|  | *Low* | 2/8 |
| **Subcellular localization in positive cases** | *Cytoplasmic* | 8/8 |
|  | *Nuclear* | 0/8 |
